# Special Issue: “Advances in Homogeneous Catalysis”

**DOI:** 10.3390/molecules25071493

**Published:** 2020-03-25

**Authors:** Joaquín García-Álvarez

**Affiliations:** Laboratorio de Compuestos Organometálicos y Catálisis (Unidad Asociada al CSIC), Departamento de Química Orgánica e Inorgánica, (IUQOEM), Centro de Innovación en Química Avanzada (ORFEO-CINQA), Facultad de Química, Universidad de Oviedo, E-33071 Oviedo, Spain; garciajoaquin@uniovi.es; Tel.: +34-985-103-464

**Keywords:** transition-metal-catalysis, organo-catalysis, bio-catalysis, homogeneous catalysis, organic synthesis

## Abstract

The use of enzymes, organo-catalysts or transition metal catalysts, as opposed to the employment of stoichiometric quantities of other traditional promoters of different organic synthetic processes (like, inorganic/organic bases, Brønsted acids, radicals, etc.) has allowed the discovery of a great number of new synthetic protocols within the toolbox of organic chemists. Moreover, the employment of the aforementioned catalysts in organic synthesis permits: (*i*) the diminution of the global energy demand and production cost; (*ii*) the enhancement of both the chemoselectivity and stereoselectivity of the global process; and (*iii*) the reduction of metal-, organo- or bio-catalyst consumption, thanks to the possible recycling of the catalysts; all these being synthetic concepts closely related with the principles of so-called *Green Chemistry***.** Thus, this Special Issue on “*Advances in Homogenous Catalysis*” has been aimed to showcase a series of stimulating contributions from international experts within different sub-areas of catalysis in organic synthesis (ranging from metal-, organo-, or bio-catalyzed organic reactions).

During recent decades, the use of metal- [1,2], organo- [3], or bio-catalysts [4] in *Organic Synthesis* has granted access to new molecules of complex structure through the formation of new carbon–carbon or carbon–heteroatom bonds, in a myriad of scientific reports. In addition, and as Gadi Rothenberg stated in his book “*Catalysis*” [5], catalysis is the key to sustainability. A catalyst is a chemical substance that facilitates a chemical reaction without changing its thermodynamic parameters, by creating a new and kinetically favored reaction pathway that is faster than the non-catalyzed version of the reaction. Moreover, the catalyst is not consumed in the process, thus, a small amount of the aforementioned catalyst can participate in several catalytic cycles, paving the pathway for the possible recyclability of the catalytic system [6]. Moreover, the employment of catalysts in organic synthesis also offers other interesting advantages, thus opening the door to the improvement of:

(i) The selectivity of a selected process, which indirectly affects its efficiency, obtaining the desired product with minor amounts of by-products and waste, thus decreasing the energy consumption and maximizing the total atom economy [7] of the process.
(ii) The tolerance to potentially degradable or sensitive functional groups, since catalytic reactions usually take place under milder reaction conditions than stoichiometric processes.
(iii) The enantiomeric excess: the employment of chiral catalysts allows to transfer the chiral information from the catalyst to the desired enantiomerically pure compounds, which usually present high added value and are very interesting for the pharmaceutical industry.

Furthermore, it is important to note that, in the field of organic synthesis, the use of metallic complexes, organocatalysts or enzymes represents one of the cornerstones of the development of new synthetic methodologies, since it not only allowed previously discovered stoichiometric organic reactions to be carried out in a catalytic manner [1,2,3,4], but also unlocked the discovery of new processes in organic synthesis that were unknown until the irruption of catalysis. Finally, it is worth noting that it is difficult to find advanced organic syntheses (both at the academic or industrial level) in which any of the aforementioned catalytic methodologies (metal-, organo- or bio-catalysis) are not used in any of their synthetic stages.

Thus, this *Special Issue* includes two original research articles and two review articles, covering different topics ranging from transition-metal-catalyzed organic transformations to ionic-liquid-mediated organic reactions. Firstly, in a communication-type article, Song, Xiao and co-workers report an efficient and selective Zn(OTf)_2_-catalyzed cascade cyclization of 2-propynol benzyl azides (**1**) with different diphenylphoshine oxides (**2**) in acetonitrile as solvent at 100 °C (see Scheme 1). The desired phosphorylated isoindolines, fused with triazoles (**3**), were obtained with moderate to excellent yields (57–91%) [8]. 

In the second original research paper, Glotov and co-workers present the synthesis of seven different polyoxometalate based-ionic-liquids (derived from pyridine or nicotinic acid, see Figure 1) and their use as catalyst in the oxidation of organosulfur compounds (like methylphenylsulfide, benzothiophene, 5-methylbenzothiophene, dibenzothiophene, 4-methyldibenzothiophene or 4,6-dimethyldibenzothiophene) in both model and real diesel fuels [9]. Moreover, the authors show that the catalytic system can operate for five consecutive cycles without any decrease in its catalytic activity.

Regarding the review-type contributions to this *Special Issue*, Guo and co-workers present a general overview of the use of cyclic carbonates and carbamates as starting building blocks in organic synthesis via transition-metal-catalyzed decarboxylation for the synthesis of different heteroaromatic organic architectures, ranging from chiral tetrahydroquinolines, functionalized indoles and furanbenzodihydropyran derivatives to chiral 3-indolin malononitriles, among others [10]. Finally, Dr. Noel Nebra reports an outstanding and complete review of high-valent Ni(III) and Ni(IV) organometallic species [11], which is nowadays a hot topic area in the field of transition-metal-catalyzed formation of C–C and C–Heteroatom bonds. Moreover, the author also presents in his review the actual state of the art on the different mechanistic proposals to support the catalytic activity of the aforementioned high-valent Ni(III) and Ni(IV) organometallic species.

In summary, the collection of original research and review articles included in this themed issue offer a broad view of the state of the art in “*Advances in Homogenous Catalysis*”, highlighting the enormous scope for advancement and application in this field. 

## Figures and Tables

**Scheme 1:**
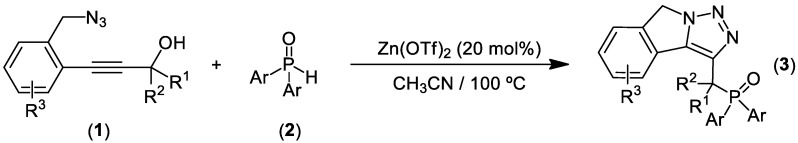
Zn(II)-catalyzed cyclization of 2-propynol benzyl azides (**1**) with different diphenylphoshine oxides (**2**), reported by Song, Xiao et al. [8].

**Figure 1:**
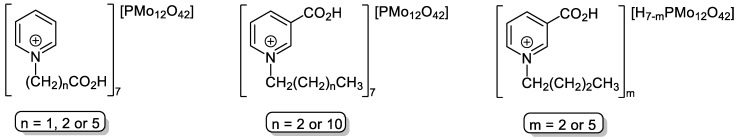
Different polyoxometalate-based ionic liquids derived from pyridine or pinacolinic acid, employed by Glotov and co-workers as catalysts for the oxidation of organosulfur compounds in both model and real diesel fuels [9].
